# Exploring photoacoustic spectroscopy-based machine learning together with metabolomics to assess breast tumor progression in a xenograft model ex vivo

**DOI:** 10.1038/s41374-021-00597-3

**Published:** 2021-04-19

**Authors:** Jackson Rodrigues, Ashwini Amin, Chandavalli Ramappa Raghushaker, Subhash Chandra, Manjunath B. Joshi, Keerthana Prasad, Sharada Rai, Subramanya G. Nayak, Satadru Ray, Krishna Kishore Mahato

**Affiliations:** 1 Department of Biophysics, Manipal School of Life Sciences, Manipal Academy of Higher Education, Manipal, Karnataka India; 2 Department of Electronics & Communication Engineering, Manipal Institute of Technology, Manipal Academy of Higher Education, Manipal, Karnataka India; 3 Department of Ageing Research, Manipal School of Life Sciences, Manipal Academy of Higher Education, Manipal, Karnataka India; 4 Manipal School of Information Sciences, Manipal Academy of Higher Education, Manipal, Karnataka India; 5 Department of Pathology, Kasturba Medical College, Mangalore, Manipal Academy of Higher Education, Mangalore, Karnataka India; 6 Department of Surgery, Kasturba Medical College, Mangalore, Manipal Academy of Higher Education, Mangalore, Karnataka India

**Keywords:** Translational research, Preclinical research

## Abstract

In the current study, a breast tumor xenograft was established in athymic nude mice by subcutaneous injection of the MCF-7 cell line and assessed the tumor progression by photoacoustic spectroscopy combined with machine learning tools. The advancement of breast tumors in nude mice was validated by tumor volume kinetics and histopathology and corresponding image analysis by TissueQuant software compared to controls. The ex vivo tumors in progressive conditions belonging to time points, day 5^th^, 10^th^, 15^th^ & 20^th^, were excited with 281 nm pulsed laser light and recorded the corresponding photoacoustic spectra in time domain. The spectra were then pre-processed, augmented for a 10-fold increase in the data strength, and subjected to wavelet packet transformation for feature extraction and selection using MATLAB software. In the present study, the top 10 features from all the time point groups under study were selected based on their prediction ranking values using the mRMR algorithm. The chosen features of all the time-point groups were then subjected to multi-class Support Vector Machine (SVM) algorithms for learning and classifying into respective time point groups under study. The analysis demonstrated accuracy values of 95.2%, 99.5%, and 80.3% with SVM- Radial Basis Function (SVM-RBF), SVM-Polynomial & SVM-Linear, respectively. The serum metabolomic levels during tumor progression complemented photoacoustic patterns of tumor progression, depicting breast cancer pathophysiology.

## Abstract (web summary)

The current study reports the assessment of tumor progression in athymic nude mice by Photoacoustic spectroscopy-based machine learning tools. The progressive tumors were classified using multi-class Support Vector Machine (SVM) algorithms with 99.5% accuracy. The serum metabolomic levels during tumor progression complemented photoacoustic spectral features, depicting breast cancer pathophysiology.

## Introduction

The most common malignancy among women worldwide is breast cancer. According to GLOBOCAN 2018, breast cancer accounts for ~2.1 million new cases and 0.6 million deaths in women worldwide due to breast cancer [1]. Breast cancer in an advanced stage with distant metastases is almost incurable with the existing therapeutic methods. Therefore, detecting breast cancer before time is the best possible treatment planning option, thereby eliminating the disease’s apparent consequence [2].

Histopathology is currently considered the gold standard for diagnosing various cancers, including breast cancer, by assessing structural changes in tissues [3]. Various researchers worldwide have made continuous attempts to find appropriate methods for diagnosing breast cancer, and many of the techniques currently being used are extensively being tested. Mammography, which uses ionizing radiation, demonstrates low sensitivity and specificity upon the increase of tissue density. Ultrasound being a low-resolution technique, sometimes cannot confirm malignancy without a biopsy. Magnetic Resonance Imaging (MRI), because of its restricted specificity, often gives false-positive results and is not suitable for in situ carcinoma. The use of Computed tomography (CT) has radiation risks, expensive as well as time-consuming. In the case of Positron Emission Tomography - Computed Tomography (PET/CT), skilled personnel to handle radioactive tracer injection is required and is not suitable for young women [4–6]. Microwave biosensor-based methods for early-stage breast cancer detection have shown the potentiality of Microwave Imaging (MI) as an alternative or additional tool to mammography. However, there are quite a few limitations to the practical application of this technique, primarily being computationally expensive. Other constraints, like penetration of microwaves in breast tissues, are dielectric property dependent, vary with frequency and temperatures. Also, a significant attenuation of the electromagnetic waves within the tissues with higher conductivity, limitation in spatial resolution, phase distortion inside and around the biological objects, etc. are other drawbacks of MI. Therefore, developing a high-sensitive and quick diagnostic technique for early-stage breast cancer is crucial [7, 8].

There are reports of using optical techniques in diagnosing malignant breast tissues [9, 10]. Optical techniques like fluorescence imaging and fluorescence lifetime imaging provide a non-destructive probing of tissue structures and composition, making them safe for intraoperative use [11]. Photoacoustics, on the other hand, is an emergent imaging modality in the biomedical field and has demonstrated its application in a variety of tissue types involving pre-clinical and clinical models [12]. The technique has been tested preclinically and clinically for the imaging of the brain, thyroid, breast as well as for dermatologic imaging [13–16], Intraoperative imaging, Lymphatic system imaging, Gynecological, and urologic imaging, etc. [17]. The technique provides high image contrast and, with the help of endogenous and exogenous contrasting agents, adequate depth of imaging. In recent times, there has been tremendous progress achieved in the instrumentation and imaging applications by the technique [18]. The photoacoustic spectroscopy’s ability to detect biochemical alterations upon the onset of breast tumor development has been demonstrated [19]. In combination with machine learning algorithms, photoacoustic spectroscopy has shown tremendous improvement in the classification of abnormal tissues from healthy, signifying its diagnostic capability and possible alternative to currently available tools for disease diagnosis [20].

The experimental data obtained from biological specimens are the multivariate types, which arise mostly from the samples’ heterogeneity nature. Various statistical approaches have been used in the past and present to extract meaningful information from such data, uncovering obscure patterns within datasets and revealing the underlying pathology and pathogenesis [21]. Among the tested statistical approaches, Machine learning is gaining more popularity because of its performance and sensitivity to data classification. Machine learning (ML) algorithms involve mathematical models applied to train data sets by adapting their parameters to predict. Machine Learning can identify distinct features from the data to be used for predicting them. The Machine Learning algorithms has broadly been used in the prognosis and monitoring of cancer by extracting the molecular features from cell-free DNA in the blood [22], also used in the classification of melanoma [23] and for detecting lymph node metastases in breast cancers from histological images [24]. However, training a machine learning model for optimal data classification typically requires large training dataset which poses a practical challenge to obtain from biological samples due to non-availability and ethical issues. Data augmentation techniques can be implemented to generate synthetic training data to address this issue of data scarcity. Data augmentation enhances the available limited dataset by transforming existing samples to create new ones [25]. This approach of augmenting data is a familiar methodology in computer-vision domain but has not been fully explored in addressing time-series classification, which is attempted in the present study on time-domain photoacoustic spectra.

For the spectral signals of various sample types, the hidden features can be extracted through spectral transformations using “Fast Fourier Transform” - FFT and wavelet transform – WT tools. Further, since an appropriate spectral feature is needed to classify data through Machine learning accurately, selecting the right spectral transformation tool for feature extraction is the key to data classification. In most real-time signals, including photoacoustic signals, WT demonstrates a better performance than FFT. This is because “FFT” does not significantly extract minor differences in the input signals compared to WT, making WT a better data transformation tool for feature extraction [26, 27]. There are many “wavelet transform” (WT) types, and they decompose signal waves onto a set of essential functions known as wavelets, obtained from a single model wavelet, called mother wavelet [28]. In WT, “Wavelet Packet Decomposition” (WPD) is used to generate both “approximations” and “details” coefficients from the input signals understudy in the low and high-frequency regions, respectively. WPD is an iterative process carried out until the desired levels are achieved. The spectral features thus extracted from various pathological samples can be used for discrimination analysis applying different machine learning tools, including Support Vector Machine (SVM) analysis [29]. SVM is an effective supervised learning method applied to data classification of different sample types used in high-throughput technologies generating a large volume of data. In the present study, to classify photoacoustic spectral data of tumor progression in the xenograft mice model, SVM learning has been used.

In the present study, upon breast tumor induction using MCF-7 cells injection to athymic nude mice evaluated for tumor progression by photoacoustic spectroscopy- a novel approach combined with machine learning tools. The tumor progression was further validated using histopathological evidence of tumor growth and corresponding quantitative image analysis by TissueQuant software compared to control. The serum metabolites analysis of the control and progressive stages of the tumor was also performed in the study, drawing a correlation of the biochemical changes with corresponding photoacoustic signatures upon tumor progression.

## Materials and methods

### Tumorigenesis and sample collection

The current study was carried out as per the guidelines and approval of the Institutional Animal Ethics Committee (IAEC), MAHE, Manipal, India. The breast tumor xenograft was developed in BALB/c, athymic nude mice (*n* = 5) using the MCF-7 cell line. According to established protocol, ~5 million of 75–85% confluent MCF-7 cells suspended in 100 μl of 100% Matrigel were injected subcutaneously on the animals’ flank region for inducing tumor xenograft model and 100 µl of 100% Matrigel alone to the control group. The animals were regularly checked for tumor development, and the tumor progression was measured for length and breadth using a digital Vernier caliper. The tumor volume kinetics was calculated using the formula mentioned below [19]. Based on the allowed tumor volume limit after MCF 7 cells injection, the entire tumor growth period was divided into 4 assessment time points (day 5, 10, 15, and 20) and conducted the study considering day 0 as control.

$${\mathrm{Tumor}}\;{\mathrm{Volume}} = \left( {{\mathrm{width}}^{\mathrm{2}}\times {\mathrm{length}}} \right)\!{{/ 2}}\;{\mathrm{mm}}^{\mathrm{3}}$$

On reaching the defined time points, the animals were anesthetized, injecting 50 mg/kg Sodium Thiopentone intraperitoneally and collecting 2 ml of blood from each through the retro-orbital puncture. Immediately after the withdrawal of blood samples, the secondary physical method of euthanasia was followed to ensure animals’ death, and tumor masses were harvested. A small piece of the tumor mass was fixed immediately in 10% Neutral buffered formalin (NBF) for histopathological study. The remaining tumor tissues were snap-frozen and stored at −80 °C for further studies. The frozen tissues were thawed to room temperature, and photoacoustic spectra were recorded.

### Photoacoustic spectral recordings

**Fig. 1:**
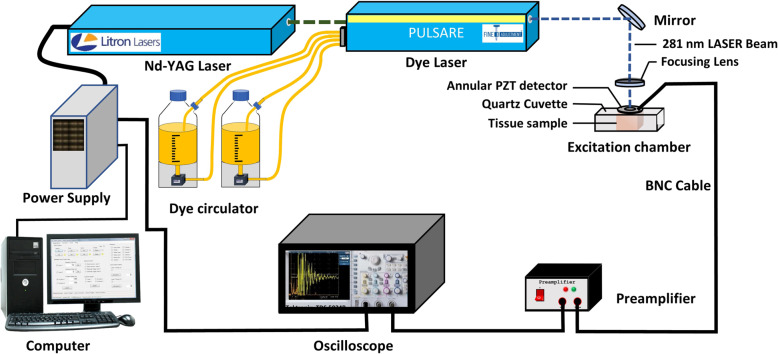
Photoacoustic instrumentation. Block diagram of the experimental setup used to record Photoacoustic spectra of tumor tissues belonging to different days of tumor progression (days 5^th^, 10^th^, 15^th^, 20^th^) and control (day 0^th^).

The photoacoustic spectra from the tumor tissues were recorded ex vivo using the experimental setup consisting of an Nd-YAG laser (LITRON Lasers, United Kingdom) pumped frequency-doubled dye laser (PULSARE Pro, FINE ADJUSTMENT, Germany) as an excitation source. The second harmonic (532 nm) of the Nd-YAG laser was used to pump the dye laser containing Rhodamine 6G dye to obtain 281 nm laser light for the samples’ excitation. The laser light (energy ≈ 100 μJ per pulse) was focused onto the tissue samples placed in a quartz cuvette of dimension: 35 mm (Height) x 10 mm (Width) x 10 mm (Depth) using a 6 mm focusing fused silica ball lens (Edmund Optics), held in contact with the PZT detector (PI Ceramics, Germany), maintaining a constant pressure throughout. As a result of 281 nm pulsed laser excitations, the induced photoacoustic signals in the tumor tissues were detected by the PZT detector, and after amplification using a preamplifier, they were recorded in the time domain on an oscilloscope (Tektronix, TDS 5034B) as shown in Fig. 1. The photoacoustic spectra were recorded from each tumor tissue in 4 different positions and 5 spectra from each location. Hence, a total of 500 photoacoustic spectra (5 spectra × 4 positions × 5 tissues × 5 groups) were recorded. The photoacoustic spectra were then processed using MATLAB R2019b (MathWorks, USA) software. The sampling frequency was set to 2.6 MHz during the photoacoustic signal acquisition.

### Data analysis

**Fig. 2:**
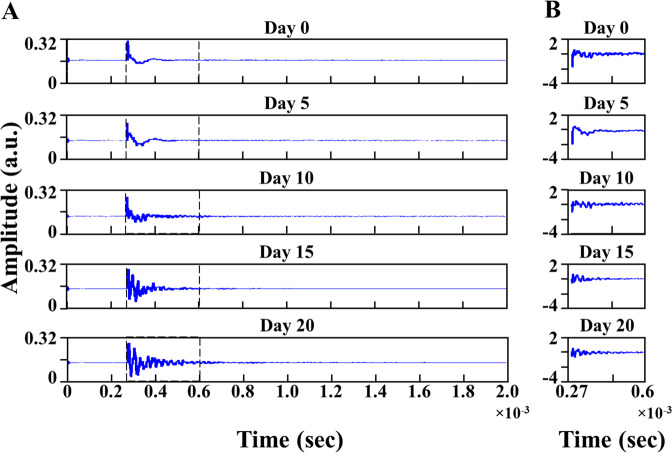
Photoacoustic spectral representation. (**A**) Typical photoacoustic spectra of tumor tissues in time-domain on different days (5^th^, 10^th^, 15^th^, 20^th^) of tumor progression and control (day 0^th^), with ROI, marked. (**B**) Typical pre-processed photoacoustic spectra in the time domain with the selected region of interest for different days (0^th^, 5^th^, 10^th^, 15^th^ and 20^th^) of tumor progression.

The typical raw photoacoustic spectra of different time point groups under study recorded in time-domain in the region 0–2 ms are shown in Fig. 2A. The raw spectra were processed for further analysis using MATLAB R2019b software (MathWorks, USA).

#### Pre-processing

Initially, we pre-processed the spectra from each sample of all the experimental groups under study. The pre-processing step involved detrending, baseline correction, background subtraction, and normalization of the spectra. After pre-processing, we selected a common ‘region of interest’ (ROI) with a maximum spectral variation for all the photoacoustic spectra under study as shown in Fig. 2B. In the present study, ROI between 0.27 and 0.6 ms along the time axis showed maximum spectral variations and was selected for further analysis.

#### Augmentation

**Fig. 3:**
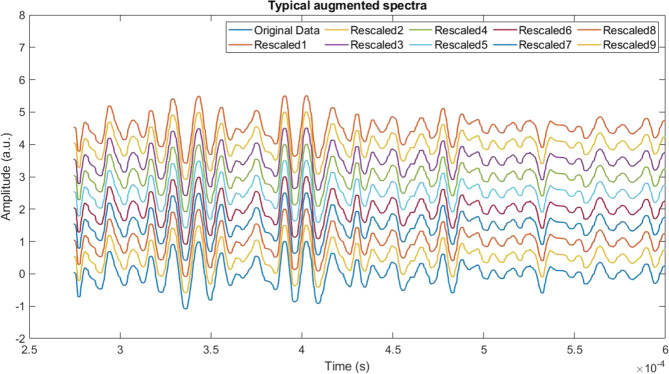
Spectral data augmentation. A typical augmentation conducted on an ROI selected photoacoustic spectrum using the process rescaling. Each spectrum was incrementally rescaled ranging from 0.5 to 4.5 in a step of 0.5 and generated 9 augmented spectra for each, making a total of 10 spectra including original.

In the present study we followed Time-series Data Augmentation. We have adopted rescaling as a data augmentation technique, which changes the raw data’s magnitude, preserving the labels. Scaling is implemented by multiplying the original data by a random scalar [25]. In the current study we have employed rescaling as data augmentation technique using 0.5–4.5 variants in an increment of 0.5 on the original photoacoustic spectra to increase 10-fold data strength as compared to original data. A typical augmented photoacoustic data obtained from a day 20^th^ photoacoustic spectrum is shown in Fig. 3. The augmented and original data were then combined together and used for further analysis.

#### Wavelet transformation

The pre-processed ROI selected photoacoustic spectra (Fig. 2B, typical spectra) along with augmented spectra were subjected to wavelet transformation using a Wavelet Packet Transform (WPT)/Wavelet Packet Decomposition (WPD) to extract the hidden spectral features from them. WPT offers optimal signal analysis given by three parameters: scale/level, position, and frequency. A pictorial representation of the WPT workflow (Binary tree) is shown in Supplementary Fig. S1. In the present study under WPT, we have used the sub-band filtering on the input spectral signals of equal widths for each decomposition level to capture the right information from the data and approximation and detail information from the spectra decomposed. Each decomposed level preserved the global/total energy and can reconstruct the exact/original spectral patterns. In the present study, the mother wavelet db6 was used as a predictive model because it demonstrated better results than others. The energy distribution of the wavelet coefficients for the level 2 decomposition for the photoacoustic signals of day 0^th^, 5^th^, 10^th^, 15^th^, and 20^th^ groups under study found to be ~99.2%, 99.8%, 99.8%, 99.9%, 99.9% respectively for approximate coefficient ‘AA2’ as shown in Supplementary Fig. S2. As depicted in Figs. S1 and S2, the optimal signal strength was observed in decomposition level 2 under frequency level (2, 0) i.e., AA2 and hence was considered the ideal decomposition level. Since the frequency of interest has maximum energy falling under AA2, it was used for further analysis.

#### Feature selection

In the present study, ‘minimal Redundancy Maximal Relevance’ (mRMR) feature selection algorithm was used to eliminate redundant features confined in the WPT coefficient (2,0). The algorithm selects the best among the features by eliminating the noise and discriminates the class better [30]. The mRMR uses an empirical method in choosing the features that are mutual and having maximum dissimilarities. This method was followed by the feature ranking based on prediction importance score. In the present study, 10 features were found optimal for the discrimination of the spectra from different groups under study and were used as an input feature matrix for the machine learning algorithms for training and testing [31].

#### Classification

The features selected using the mRMR algorithm from the combined original and augmented photoacoustic spectral data of different time point groups (day 0^th^, 5^th^, 10^th^, 15^th^ & 20^th^) under study were fed to the SVM learning algorithm for training and classification to their respective time points groups. The feature matrix contained 10 features belonging to 5000 photoacoustic spectra, 500 original spectra, 100 belonging to each of 5 time points groups (day 0^th^, 5^th^, 10^th^, 15^th^ & 20^th^) plus 4500 augmented spectra obtained with 9 augmentations on each spectrum. A multi-class SVM learning model (RBF, Polynomial and Linear) trained using 80% of the feature matrix data belonging to each group under study and 20% used for testing of the model.

These SVM algorithms work on both linear and nonlinear modes of classification. It separates the classes using hyperplanes, created by forming support vectors and margins. The support vectors are the data points that are closer to the hyperplanes. Margin is the maximum value of the separation of the hyperplanes of different classes. The hyperplane position depends on the locus of the support vectors. There are different kinds of kernel functions; ‘Radial Basis function’ (RBF), ‘Polynomial’, ‘Linear’, and ‘Sigmoid’ used in SVM, ‘RBF’ and ‘Polynomial’ are used for nonlinear classifications. In the present study, we have used SVM-RBF, SVM-Polynomial, and SVM-linear models to train and test the data. The models’ performance was evaluated by ‘Specificity’, ‘Sensitivity’ & ‘Accuracy’. For SVM, the classification score for a sample value (x) is the signed distance from x to the decision boundary, ranging from −∞ to + ∞. The highest score value indicates that it belongs to the target class and a negative score indicates that it belongs to another category. The predicted score is given by the function, as below,

$$\hat f\left( x \right) = \mathop {\sum }\limits_{j = 1}^n \alpha _jy_jG(x,x_j) + \hat b$$

where ($\alpha _1,\alpha _2,$…., $\alpha _n,b$) is the estimated SVM parameters which are obtained by the trained model, G($x_j,x$), known as the dot product between x and the support vectors in the predictor region for the training set of the sum of observations [29].

### Histology

The formalin-fixed tumor tissues were dehydrated with graded ethanol (50–100%), further defatted in xylene, and embedded in paraffin. Subsequently, a 5 μm thick section of the paraffin-embedded tissues were used for Hematoxylin and Eosin (H&E) staining. The paraffin sections were deparaffinized and were rehydrated and stained with hematoxylin solution for 5 min. This step was followed by 1 dip in 1% acid alcohol solution (1% HCl in 70% ethanol), the excess stain was washed in tap water. Afterward, the sections were stained using eosin solution for 3 min and dehydrated with graded alcohol and cleared in xylene [32].

### Liquid chromatography-mass spectrometry (LCMS)

The metabolomic analysis was performed using Agilent 6520 Accurate-Mass Q-TOF LC/MS System with Agilent 1200 Series HPLC unit (Agilent Technologies, Santa Clara, California, USA). The blood serum samples obtained from the experimental animals of various time-point groups were used for the metabolomic analysis. The metabolites were extracted by mixing the serum and ice-cold methanol in 1:2 ratio, followed by vortexing the mixture for 15 min. This was centrifuged at about 12,000 rpm, 12 min. The supernatant collected was lyophilized, and the residue was dissolved with 0.1% formic acid in 30 μl of 5 % acetonitrile (95:5). An 8 μl of the processed serum samples from different experimental groups under study were injected into the reversed-phase column (ZORBAX Eclipse Plus C18, 4.6 mm × 150 mm, 5 µm; Agilent Technologies). Metabolites were separated using a gradient system in LC maintained by Solvent A (0.1% formic acid in water) and B (0.1% formic acid in 90% acetonitrile). Basic and neutral metabolites were eluted in positive mode using a gradient flow rate of 400 µl/min in mobile phase A and mobile phase B (92% A & 8% B in 25 min, 8% A & 92% B for 10 min and equilibrated to 92%A & 8% B for 10 min) [33].

The raw data obtained from each run were processed using the Qualitative Mass Hunter Analysis Software B.07.00 (Agilent Technologies, Santa Clara, California, USA), a molecular feature extraction tool. The data files containing monoisotopic mass, respective abundance, and retention time were used for data alignment and filtering under Mass Profiler Plus software (MPP) (Agilent Technologies, version B.12.5). Features that were present in at least 60% of the sample numbers (3 out of 5) in each group were considered for further analysis. Compounds were identified in METLIN and HMDB database based on isotopic pattern distribution and accurate mass within a specified tolerance (15 ppm error). Fragmentation patterns of metabolites were matched with in-house developed MS/MS spectral library. The intensity values of the identified metabolites from the control and test groups were log10 transformed and used to plot a circular bar graph using CIRCOS. Metabolites were then subjected to one-way ANOVA for multiple groups in the study. Subsequently, Metabolomic Pathway Analysis (MetPA) was carried out using MetaboAnalyst software 4.0.

### Statistical analysis

The results are expressed as the mean ± standard error (SEM), and Student’s *t* test and one-way ANOVA performed for statistical significance. A *p* value < 0.05 (*n* = 5) was considered significant. The statistical analyses were carried out using GraphPad Prism 8.0 software (San Diego, CA, USA).

## Results

### Tumorigenesis

**Fig. 4:**
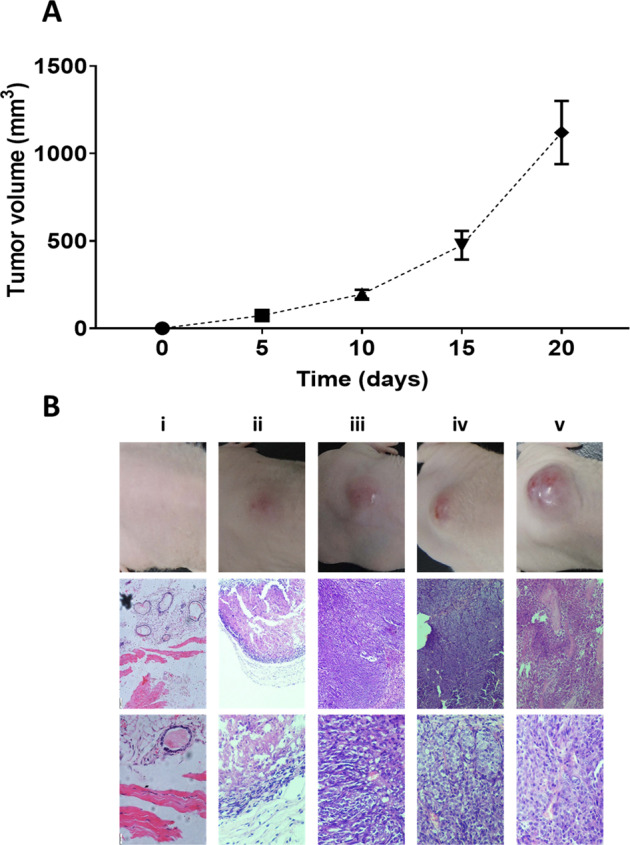
Tumor volume kinetics and histology. (**A**) Plot of tumor kinetics showing progression of tumor volume using mean tumor volumes at days 5^th^, 10^th^, 15^th^ and 20^th^ post- MCF-7 cells inoculation in athymic nude mice as compared to day 0 control. (**B**) Pictures of the intact breast tumor (i–v; top row) and the corresponding H & E stained sections in 100x magnification (i–v; middle row) and 400x magnification (i–v; bottom row) for different time point groups (days 0^th^, 5^th^, 10^th^, 15^th^ & 20^th^; i–v) of tumor progression post-MCF-7 cells injection, along with day 0^th^ control.

In the study, the tumor xenograft in athymic nude mice was established using MCF-7 cell lines. In the 5th day of post-MCF-7 cells injection, a small palpable tumor was observed, and the tumor volume showed an exponential growth in subsequent days till day 20, reaching the maximum permissible tumor volume limit of 1000 mm^3^. The control group has shown no growth at the site of injection. By considering the tumor volume of the control group as 0 mm^3^, the statistical analysis, as mentioned before, was performed. The data obtained from tumor volume kinetics were plotted, demonstrating the progressive tumor condition shown in Fig. 4A.

### Histology

The histological findings in the study showed different tumor development stages, as shown in Fig. 4, depicting the picture of intact tumor in nude mice (Fig. 4B-top row) and H&E stained images in 100x and 400x (Fig. 4B – middle & bottom row), respectively. On day 5^th^, approximately one-fifth of the tumor cells were found viable. But as the tumor progressed, a better establishment of the tumor was seen on day 10^th^, showing more viable cells with few blood capillaries. Day 15^th^ showed well-developed blood capillaries compared to the former stage and viable tumor cells. The day 20^th^ histological finding showed well-developed blood capillaries, and, in some regions, extensive necrosis was also observed.

### Photoacoustic study

**Fig. 5:**
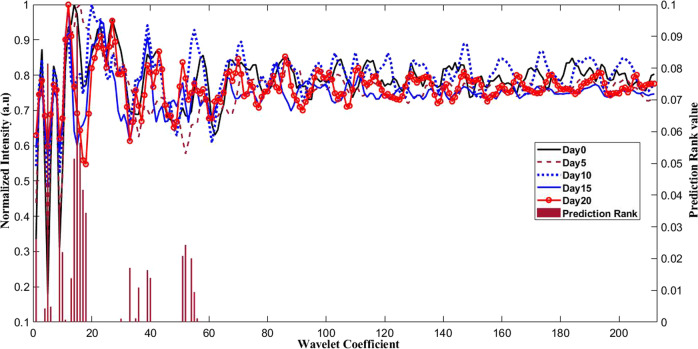
Mean photoacoustic spectra & prediction ranking. Mean photoacoustic spectra in the region of interest (ROI) from day 0^th^, 5^th^, 10^th^, 15^th^ & 20^th^, after WPD (line graph)- a time versus amplitude plot. The bar graph represents the features after mRMR performed on the data- a plot of wavelet coefficient versus prediction rank values.

**Fig. 6:**
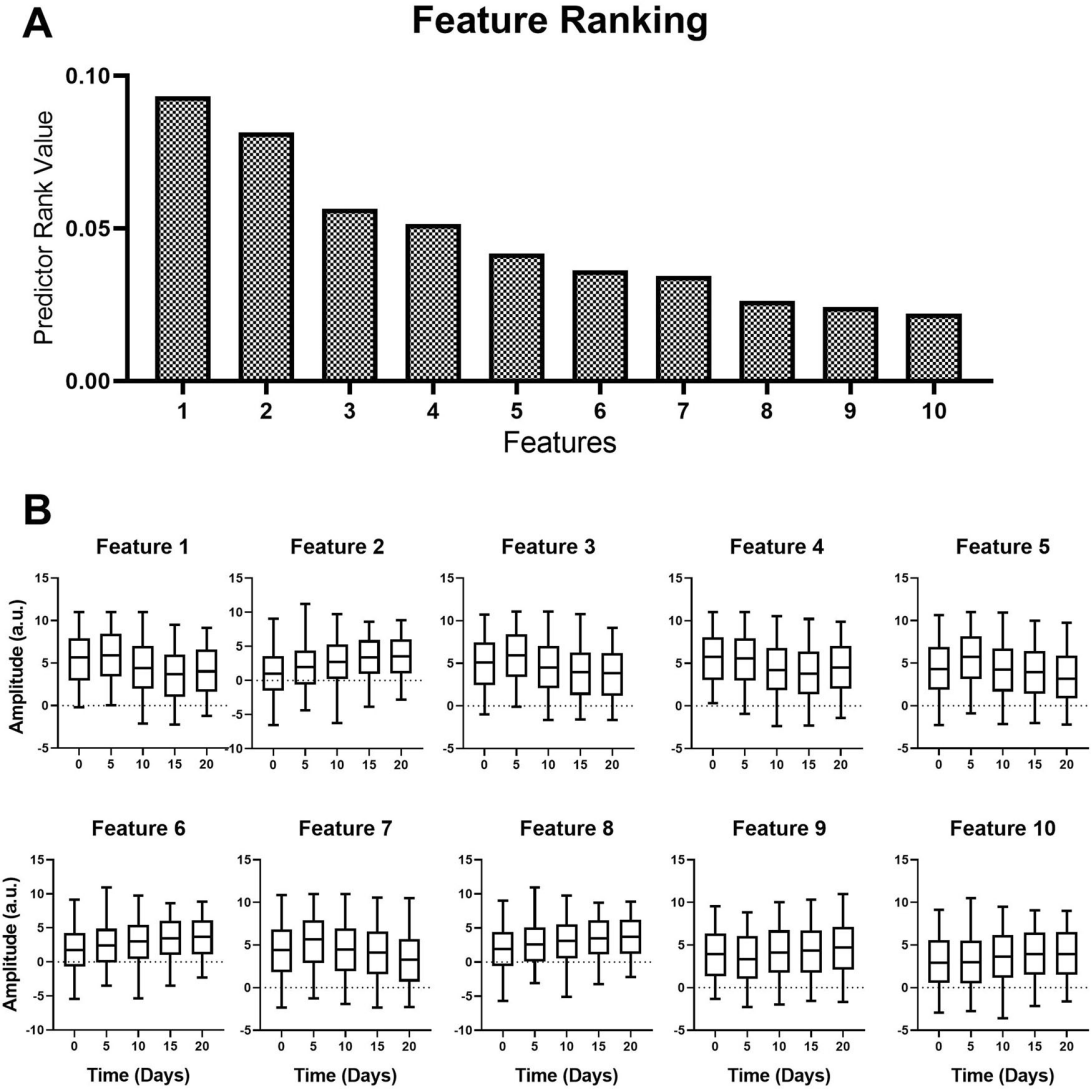
Selected features for machine learning as input matrix. (**A**) Top 10 features and their predictor importance score. (**B**) Coefficients of the top 10 selected features for individual groups used as input feature matrix for SVM learning. One-way analysis of variance test predicted *p* < 0.0001(***) and Dunnett test for multiple pairs significant with *p* < 0.0001.

A differential photoacoustic spectral pattern was observed for different experimental groups under study. The mean pre-processed photoacoustic spectra of each group after WPT demonstrated varied amplitude along the time axis. The wavelet transformed spectra subsequently subjected to the mRMR algorithm and the top 10 features necessary for the discrimination of all the spectra belonging to different groups based on their prediction ranking values were obtained by the analysis, as shown in Fig. 5. As can be seen from the figure, the best feature is the feature 1 with a maximum prediction score value of 0.093 at wavelet coefficient level **15**. The rest of the features were of lesser prediction scores. These were ranked from all groups as follows; feature 2 – coefficient level 5, prediction score 0.081; feature 3 – coefficient level 16, prediction score 0.056; feature 4 – coefficient level 14, prediction score 0.051; feature 5 – coefficient level 17, prediction score 0.041; feature 6 – coefficient level 9, prediction score 0.036; feature 7 – coefficient level 18, prediction score 0.034; feature 8 – coefficient level 1, prediction score 0.026; feature 9 – coefficient level 52, prediction score 0.024; feature 10 – coefficient level 10, prediction score 0.022, as shown in Fig. 5. The variations in the features for all the time point groups under study were visualized by plotting them using GraphPad prism 8.0, which is shown in Fig. 6 along with their prediction scores. These top 10 features from all the time points groups were fed to the machine learning algorithm as an input feature matrix for classification by using multi-class SVM-RBF, SVM-Polynomial, and SVM- Linear algorithms with 80% data for training and 20% for testing. The random training and testing of the classification models were done by repeating the process 10 times and selecting best classification model for the analysis as shown in Supplementary Fig. S3. The sensitivity, specificity, and accuracy for the best classification by the models were also calculated, as listed in Table 1. The sensitivity values for classification using SVM-RBF, SVM-polynomial and SVM-linear for the 5^th^, 10^th^, 15^th^ and 20^th^ day groups are found to be 98%, 98%, 90.5%, 92.5%; 100%, 99.5%, 100%, 98% and 98%, 64.5%, 73.5%, 83.5% respectively. Similarly, the specificity and accuracy values for the analysis using SVM-RBF, SVM-Polynomial, and SVM-Linear are found to be 97%, 100%, 92% and 95.2%, 99.5%, 80.3% respectively.

**Table 1:** Performance metrics of testing 20% of the photoacoustic data for each time point group under study using SVM analysis after the training with 80% of the data from each group.

Class	SVM model	Specificity (%)	Sensitivity (%)	Accuracy (%)
Day 0	RBF	97	–	95.2
Day 5		–	98	
Day 10		–	98	
Day 15		–	90.5	
Day 20		–	92.5	
Day 0	Polynomial	100	–	99.5
Day 5		–	100	
Day 10		–	99.5	
Day 15		–	100	
Day 20		–	98	
Day 0	Linear	92	–	80.3
Day 5		–	98	
Day 10		–	64.5	
Day 15		–	73.5	
Day 20		–	83.5	

### Metabolomics

**Fig. 7:**
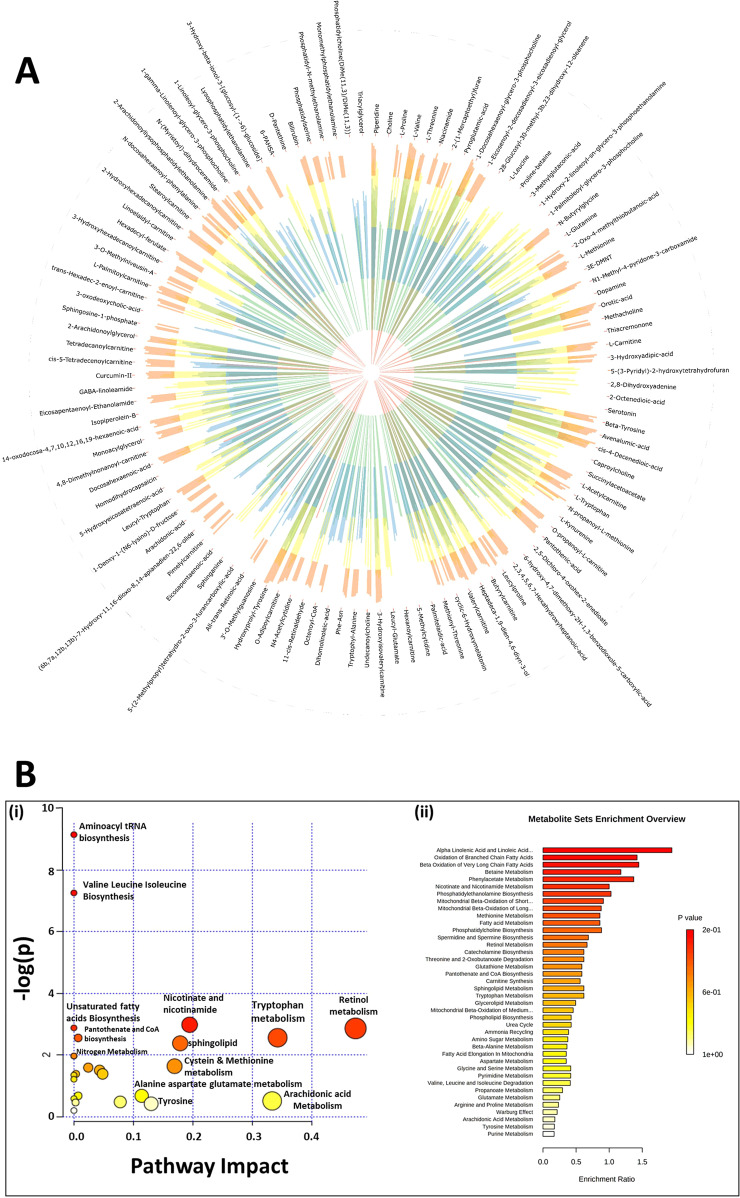
Metabolome overview and pathway analysis. (**A**) An overview of serum metabolome patterns in control and Days 5^th^, 10^th^, 15^th^, and 20^th^ groups. Circos diagram illustrates a comparison of log10 transformed intensity values of 114 identified metabolites (excluding xenobiotics) detected in control (Inner-red), Day 5^th^ (green), Day 10^th^ (blue), Day 15^th^ (yellow), and day 20^th^ (orange). (**B**) (i) Metabolic Pathway Analysis (MetPA). All the pathways are represented as circles. The color and size of each circle are based on the *p* value and pathway impact value, respectively. The plot is showing the −log of p values from the pathway enrichment analysis on the y-axis and the pathway impact values derived from the pathway topology analysis on the x-axis. (ii) Metabolic Set Enrichment Analysis showing the highly altered metabolisms during breast tumor progression. Color intensity (white-to-red) indicates increasing statistical significance and varying circle diameter with pathway impact. The graph showing −log of *p* values from pathway enrichment analysis on the y-axis the and the pathway impact values derived from pathway topology analysis on the x-axis.

LC-MS data analysis of serum samples obtained from the control and test groups under study revealed the presence of a total of 3997 spectral features/metabolites. The data was filtered to their intensity, present in a minimum of 3 out of 5 animals (60%) per group. These metabolites were annotated using the m/z ratio in METLIN and HMDB databases within 15 ppm tolerance. There were 114 compounds annotated, excluding xenobiotics and drugs. Further were used the intensity of the identified compounds to visualize the global metabolome of control and test groups using Circos illustration, as shown in Fig. 7A. The Circos representation showed differential distribution patterns across the control and test groups. Intensity and presence of metabolites were considered in each sample of all the groups, and upon performing ‘Student’s *t* test’ and ‘One-way ANOVA’ on the annotated compounds, found that 19 metabolites present in all the experimental groups were altered as shown in Supplementary Fig. S4. The metabolites were subjected to MS/MS analysis to find the fragmentation patterns to validate the annotated compounds.

The significantly altered compounds were subjected to fold change analysis. The fold change was calculated for days 5^th^, 10^th^, 15^th^ & 20^th^ groups with respect to control, as shown in the Supplementary Fig. S5. The fold change analysis showed that the serum levels of most of the metabolites to be downregulated as the tumor progressed with respect to control. Further, the metabolites from all the time-points were subjected to ‘Pearson’s correlation analysis,’ as shown in the Supplementary Fig. S6. The differentially altered serum metabolites in control and test groups were detected in serum samples based on the most abundant and significantly altered intensities and were transformed to log10 and represented as Box-Whiskers plots. Statistically significant changes in metabolite intensity between the control and day 20^th^  group are represented by asterisk (***p < 0.001, **p < 0.01, *p < 0.05). However, some metabolites showed no statistical significance compared to control, as shown in Supplementary Fig. S4.

Among 114 identified compounds, the amino acid levels, lipid levels, and other compounds showed significant variations in the current study. The student’s *t* test was performed for day 0 versus day 20^th^ group, the amino acids, threonine, proline-betaine, L-valine, tryptophan, tyrosine, and other metabolites such as 5-methylcytidine, serotonin, piperidine and propionyl choline were found to be significantly decreased in progressive tumor condition compared to control. Further, carnitine and o-propanoyl-L-carnitine also showed similar intensity trend upon tumor progression to day 20^th^. However, metabolites such as acylcarnitine, valeryl carnitine, pantothenic acid, succinyl acetoacetate and 5-(3-Pyridyl)-2-hydroxytetrahydrofuran had altered intensities upon tumor progression, nevertheless, did not show significance in downregulation.

### Pathway analysis

The annotated metabolites (114 compounds) were subjected to pathway analysis using MetaboAnalyst 4.0. It was observed that most of the pathways were affected. Among them, the tryptophan pathway was also impacted with 3 hits (tryptophan, serotonin & kynurenine) with a raw *p* value of 0.0443 and a pathway Impact of 0.34, respectively. The graphical representation of pathway analysis is shown in Fig. 7B with the list of pathways impacted.

## Discussion

In the present study, breast tumor progression was assessed by photoacoustic spectroscopy combined with machine learning using support vector machine analysis. Initially, the tumor induced as mentioned in the materials and methods was validated by tumor volume kinetics (Fig. 4A) and histological study (Fig. 4B), also explained in the Supplementary Section 1. Tumor progression on day 0, day 5, day 10, day 15, and day 20 post tumor induction, was also validated by histological image processing, as explained in Supplementary Section 4. Photoacoustic spectroscopy, which can detect minor biochemical changes in the biological samples, was then used to record spectral profiles of the progressive tumor conditions [34]. As mentioned in the materials and methods, there were 500 original photoacoustic spectra, 100 from each 5-time point group recorded. After pre-processing and selecting ROI containing maximum variation with tumor progression, the original spectra were augmented 10 folds (5000 spectra, 1000 from each group), the original and augmented spectral data analyzed using an SVM algorithm to predict functional outcomes of disease diagnoses [35]. Support vector machine has widely been used, replacing some of the commonly used logistic functions like SoftMax as a classifier in deep learning [36]. In cancer classification, SVM has been used as a classifier on high throughput microarray gene expression [37]. Moler et al. applied SVM in a colon cancer tissue classification using microarray gene expression data. When the performance of SVM was compared with the naive Bayes classifier using different numbers of selected genes, the former was found to outperform the latter [38]. The SVM is thus most suitable for tissue classification on a wide range of data types [39].

In the present study, to increase the data diversity and size for limiting overfitting in machine learning, all 500 ROI-selected photoacoustic spectra were subjected to data augmentation. In a study, Rashid et al., 2019 have shown a machine learning-based framework for identifying construction equipment activities to assess productivity, safety, and environmental impact on construction sites using the time-series data [25]. The study showed the impact of augmentation on the machine learning model. In the present study, data augmentation was done by adopting the option rescaling to achieve 10-fold increase in the data strength. The combined original and augmented data were subjected to Wavelet Transformation (WT) to extract hidden spectral features from them. We selected WT over FFT for feature extraction because FFT does not significantly extract minor variations from the input signals like real-time photoacoustic signals compared to WT [27]. Further, there are many WT types, and they decompose signal waves onto a set of essential functions known as wavelets, obtained from a single model wavelet, called mother wavelet [28]. In this study, we have used db6 wavelet, as mother wavelet. In WT, Wavelet Packet Decomposition (WPD) generates both ‘approximations’ and ‘details’ coefficients from the input data understudy in the low and high-frequency regions, respectively, to create the full binary tree, as shown in Supplementary Fig. 1. WPD is an iterative process, repeats until a desired level is achieved. It provides an actual signal variation required for the analysis under a particular sub-band in the process. In the present study, AA2 (2,0) sub-band demonstrated optimal signal variation and was used to select the spectral features for the analysis. WPD has also been used to classify ECG signals, providing significantly detailed signal decomposition hence improving the time-frequency resolution of the signal [26].

After the transformation of the photoacoustic spectra, feature extraction, which plays a vital role in deciding the performance of machine learning by accurately classifying the data, selecting the right spectral transformation tool for feature extraction is the key to data classification. The reduced redundancy in data contributes to the reduced noise-based prediction and results in better classification accuracy [40]. Therefore, the feature selection algorithm, the minimal Redundancy Maximal Relevance (mRMR) algorithm, is faster and computationally feasible and does not involve learning was used. The mRMR minimizes the correlation between different features of a target class in the analysis, ensuring feature ranking based on mutual information and prediction ranking, as shown in Fig. 5. As mentioned earlier, 10 features were selected by mRMR and used for data classification. Previously, mRMR based feature selection was used in microarray data analysis for determining biologically relevant genes in cancer samples. Genes selected by mRMR algorithms showed a better characteristic of phenotypes and improved prediction [30].

In the present study, the spectral features selected by mRMR were subjected to Machine Learning using Support Vector Machine (SVM) analysis to classify the photoacoustic spectral data of progressive tumor stages. SVM is an effective supervised learning method applied for data classification of different sample types in high-throughput technologies generating a large volume of data [29]. In our study, the top 10 features selected by mRMR were used as an input feature matrix for classification using multi-class SVM learnings. The multi-class SVM models used were SVM-RBF, SVM-Polynomial, SVM- Linear algorithms, and varied classification accuracy of 95.2%, 99.5% & 80.3%, respectively, were obtained. Our analysis demonstrated the best results in SVM-Polynomial compared to the rest, as shown in Table 1 and Supplementary Fig. S3. The performance metrics of the machine learning models, the sensitivity values for days 5^th^, 10^th^, 15^th^, and 20^th^ groups using SVM-RBF are 98%, 98%, 90.5%, 92.5%, respectively, using SVM-Polynomial are 100%, 99.5%, 100%, and respectively and using SVM-Linear are 98%, 64.5%, 73.5%, and 83.5% respectively. The specificity values of the analysis using SVM-RBF, SVM-Polynomial, and SVM-Linear are found to be 97%, 100%, and 92%, respectively. These outcomes are also listed in Table 1.

**Fig. 8:**
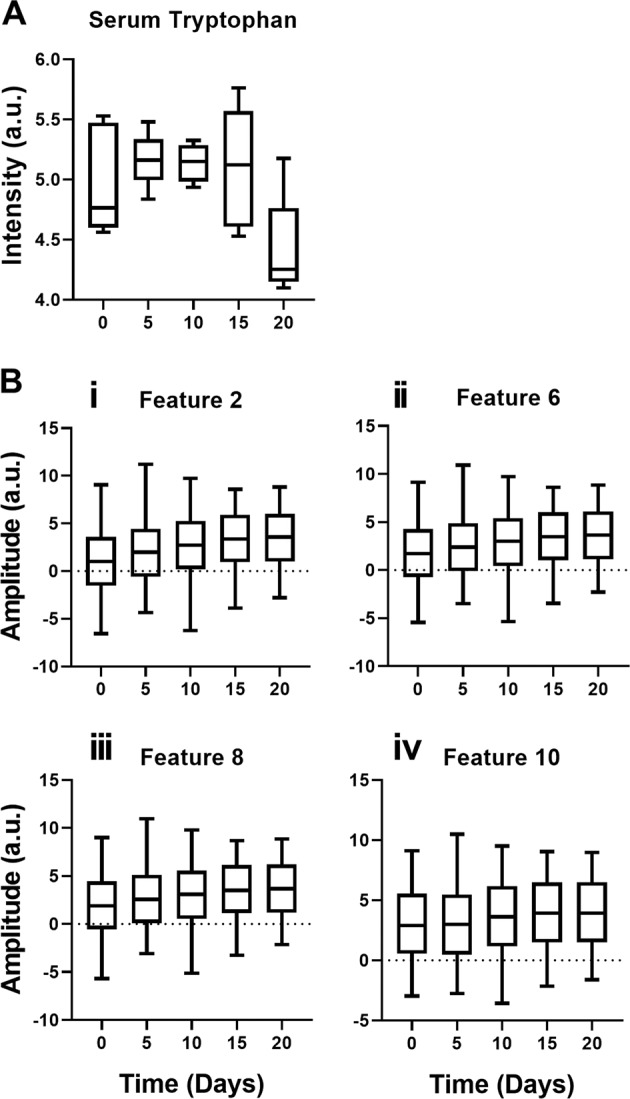
Correlation between metabolomic analysis and photoacoustic measurements of tumor progression. (**A**) Tryptophan intensity found in Day 0^th^, 5^th^, 10^th^, 15^th^, and 20^th^ obtained from serum metabolomics data analysis and t-test performed between day 0^th^ and day 20^th^ predicted *p* value as significant, *p* = 0.0109 (*). (**B**) wavelet coefficients of the features extracted from the photoacoustic signals of tumor tissues belonging to Day 0^th^, 5^th^, 10^th^, 15^th^, and 20^th^. (i) Feature 2, (ii) Feature 6, (iii) Feature 8 and (iv) Feature 10. One-way analysis of variance test predicted *p* value as significant, *p* < 0.0001(***).

In the present study, photoacoustic signals were from the tissue samples of progressive tumor conditions based on tryptophan excitation. It was also observed that spectral features 2, 6, 8, and 10 extracted from photoacoustic patterns shown increased wavelet coefficients with the tumor progression. This may be due to the uptake of tryptophan by tumor tissues and contributing to corresponding photoacoustic spectra, as shown in Fig. 8A. Further, the metabolomic analysis conducted in the present study using MetaboAnalyst software revealed tryptophan metabolism, one of the most dysregulated pathways with tumor progression. The altered serum tryptophan levels showed a decreasing trend (Fig. 8B) as the tumor progressed compared to the control group, which has also been explained in the Supplementary Data (Section 3). The decrease in serum tryptophan levels at progressive time points demonstrated an inverse correlation with spectral features 2, 6, 8, 10 in the study. This decrease in serum tryptophan and the corresponding increase in wavelet coefficients from photoacoustic measurements may be due to the uptake of tryptophan from serum by tumor tissues.

In conclusion, the current study reported an establishment of MCF-7 cells induced breast tumor xenograft in athymic nude mice and assessed tumor progression by a novel integrated approach of photoacoustics spectroscopy and machine learning. Breast tumor advancement in nude mice was also validated through tumor tissue histology in the study. Metabolomics data analysis performed in the study revealed lesser tryptophan levels in serum than tumor tissues, as reported by photoacoustic spectroscopy studies on tumor tissues. The decrease in serum tryptophan levels may be due to higher uptake of tryptophan by the tumor. Further, photoacoustic spectral analysis of tumor tissues involving WPT and mRMR for classification applying multi-class SVM-RBF, SVM-Polynomial, and SVM- Linear algorithms demonstrated varied classification accuracy of 95.2%, 99.5% & 80.3%, respectively. The SVM-polynomial showed the highest classification accuracy in the study indicating photoacoustic spectroscopy combined with machine learning could be a potential prognostic tool for assessing breast tumor progression. As per our knowledge, the present study is the first to use photoacoustic spectroscopy and machine learning for evaluating breast tumor progression supported by serum metabolomic analysis. This approach holds a very high potential for clinical translation; however, upon further modifications/improvements, the method can be brought into the clinical setting for early detection of breast cancer conditions.

## Supplementary information

Supplementary Material

## Data Availability

Data will be available upon request after the execution of a data use agreement.

## References

[1] Bray F, Ferlay J, Soerjomataram I, Siegel RL, Torre LA, Jemal A (2018). Global cancer statistics 2018: GLOBOCAN estimates of incidence and mortality worldwide for 36 cancers in 185 countries. CA. Cancer J. Clin..

[2] Harbeck N, Penault-Llorca F, Cortes J, Gnant M, Houssami N, Poortmans P (2019). Breast cancer. Nat. Rev. Dis. Prim..

[3] Makki J (2015). Diversity of breast carcinoma: histological subtypes and clinical relevance. Clin. Med. Insights Pathol..

[4] Lobbes MBI, Smidt ML, Houwers J, Tjan-Heijnen VC, Wildberger JE (2013). Contrast enhanced mammography: techniques, current results, and potential indications. Clin. Radiol..

[5] Hooley RJ, Scoutt LM, Philpotts LE (2013). Breast. Radiology.

[6] Schneble EJ, Graham LJ, Shupe MP, Flynt FL, Banks KP, Kirkpatrick AD (2014). Future directions for the early detection of recurrent breast cancer. J. Cancer..

[7] Wang L (2018). Microwave sensors for breast cancer detection. Sensors..

[8] Islam MT, Mahmud MZ, Islam MT, Kibria S, Samsuzzaman M (2019). A low cost and portable microwave imaging system for breast tumor detection using UWB directional antenna array. Sci. Rep..

[9] Godavarty A, Rodriguez S, Jung YJ, Gonzalez S (2015). Optical imaging for breast cancer prescreening. Breast Cancer Targets Ther..

[10] Akers WJ, Edwards WB, Kim C, Xu B, Erpelding TN, Wang LV (2012). Multimodal sentinel lymph node mapping with single-photon emission computed tomography (SPECT)/computed tomography (CT) and photoacoustic tomography. Transl. Res..

[11] Phipps JE, Gorpas D, Unger J, Darrow M, Bold RJ, Marcu L (2017). Automated detection of breast cancer in resected specimens with fluorescence lifetime imaging. Phys. Med. Biol..

[12] Kamath SD, Ray S, Mahato KK (2011). Photoacoustic spectroscopy of ovarian normal, benign, and malignant tissues: a pilot study. J. Biomed. Opt..

[13] Yang X, Wang LV (2008). Monkey brain cortex imaging by photoacoustic tomography. J. Biomed. Opt..

[14] Lee YJ Lee, Jeong EJ, Song HW (2017). Photoacoustic imaging probe for detecting lymph nodes and spreading of cancer at various depths. J. Biomed. Opt..

[15] Heijblom M, Piras D, Engh FM, Van D, Van M (2016). The state of the art in breast imaging using the Twente Photoacoustic Mammoscope: results from 31 measurements on malignancies. Eur. Radiol..

[16] Zhou Y, Tripathi SV, Rosman I, Ma J, Hai P, Linette GP (2017). Noninvasive determination of melanoma depth using a handheld photoacoustic probe. J. Invest. Dermatol..

[17] Steinberg I, Huland DM, Vermesh O, Frostig HE, Tummers WS, Gambhir SS (2019). Photoacoustic clinical imaging. Photoacoustics..

[18] Zackrisson S, SMWY VanDeVen, Gambhir SS (2014). Light in and sound out: Emerging translational strategies for photoacoustic imaging. Cancer Research..

[19] Priya M, Rao BSS, Chandra S, Datta A, Nayak SG, Mahato KK (2015). Monitoring breast tumor progression by photoacoustic measurements: a xenograft mice model study. J. Biomed. Opt..

[20] Allman D, Reiter A, Bell MAL (2018). Photoacoustic source detection and reflection artifact removal enabled by deep learning. IEEE Trans. Med. Imaging..

[21] Su C, Tong J, Wang F (2020). Mining genetic and transcriptomic data using machine learning approaches in Parkinson’s disease. npj Park. Dis..

[22] Cristiano S, Leal A, Phallen J, Fiksel J, Adleff V, Bruhm DC (2019). Genome-wide cell-free DNA fragmentation in patients with cancer. Nature..

[23] Esteva A, Kuprel B, Novoa RA, Ko J, Swetter SM, Blau HM (2017). Dermatologist-level classification of skin cancer with deep neural networks. Nature..

[24] Ehteshami Bejnordi B, Veta M, Johannes van Diest P, van Ginneken B, Karssemeijer N, Litjens G (2017). Diagnostic assessment of deep learning algorithms for detection of lymph node metastases in women with breast cancer. JAMA.

[25] Rashid KM, Louis J (2019). Times-series data augmentation and deep learning for construction equipment activity recognition. Adv. Eng. Informatics..

[26] Alickovic E, Kevric J, Subasi A (2018). Performance evaluation of empirical mode decomposition, discrete wavelet transform, and wavelet packed decomposition for automated epileptic seizure detection and prediction. Biomed. Signal Process. Control..

[27] Akin M (2002). Comparison of wavelet transform and FFT methods in the analysis of EEG signals. J. Med. Syst..

[28] Rhif M, Ben Abbes A, Farah I, Martínez B, Sang Y (2019). Wavelet transform application for/in non-stationary time-series analysis: a review. Appl. Sci..

[29] Joachims T. Text categorization with Support Vector Machines: Learning with many relevant features. In: Nédellec C., Rouveirol C. (eds). Machine Learning: ECML-98. 1998;1398:137–42.

[30] Ding C, Peng H (2005). Minimum redundancy feature selection from microarray gene expression data. J. Bioinform. Comput. Biol..

[31] Reiter A, Lediju, Bell MA (2017). A machine learning approach to identifying point source locations in photoacoustic data. Proc. SPIE. Photons Plus Ultrasound Imaging Sensing..

[32] Cardiff RD, Miller CH, Munn RJ (2014). Manual hematoxylin and eosin staining of mouse tissue sections. Cold Spring Harb. Protoc..

[33] Joshi MB, Pai S, Balakrishnan A, Bhat M, Kotambail A, Sharma PSVN (2019). Evidence for perturbed metabolic patterns in bipolar disorder subjects associated with lithium responsiveness. Psychiatry Res..

[34] Teo YH, Lim ICZY, Tseng FS, et al. Predicting clinical outcomes in acute ischemic stroke patients undergoing endovascular thrombectomy with machine learning. Clin. Neuroradiol. 2021. 10.1007/s00062-020-00990-3. 10.1007/s00062-020-00990-3 33491132

[35] Tang Y. Deep learning using linear support vector machines. arXiv.org. 2013. Available at https://arxiv.org/abs/1306.0239.

[36] Golub TR, Slonim DK, Tamayo P, Huard C, Gaasenbeek M, Mesirov JP (1999). Molecular classification of cancer: class discovery and class prediction by gene expression monitoring. Science..

[37] Moler EJ, Chow ML, Mian IS (2000). Analysis of molecular profile data using generative and discriminative methods. Physiol. Genom.

[38] Huang S (2018). Applications of support vector machine (SVM) learning in cancer genomics. Cancer Genom Proteomics.

[39] Li H, Yuan D, Ma X, Cui D, Cao L (2017). Genetic algorithm for the optimization of features and neural networks in ECG signals classification. Sci. Rep..

[40] Hira ZM, Gillies DF (2015). A review of feature selection and feature extraction methods applied on microarray data. Adv. Bioinform.

