# Supplementary material for: Exploring photoacoustic spectroscopy-based machine learning together with metabolomics to assess breast tumor progression in a xenograft model ex vivo
Source: Lab Invest. 2021 Apr 19;101(7):952–65. doi: 10.1038/s41374-021-00597-3 (PMC8214996; doi:10.1038/s41374-021-00597-3)
Supplement: Supplementary file 1 — Supplementary Material [file 41374_2021_597_MOESM1_ESM.docx]

**Exploring photoacoustic spectroscopy-based machine learning together with metabolomics to assess breast tumor progression in a xenograft model *ex vivo***

Jackson Rodrigues^1^, Ashwini Amin^2^, Chandavalli Ramappa Raghushaker^1^, Subhash Chandra^1^, Manjunath B Joshi^3^, Keerthana Prasad^4^, Sharada Rai^5^, Subramanya G Nayak^2^, Satadru Ray^6^ & Krishna Kishore Mahato^1^*

1. Department of Biophysics, Manipal School of Life Sciences, Manipal Academy of Higher Education, Manipal - 576104, Karnataka, India
2. Department of Electronics & Communication Engineering, Manipal Institute of Technology, Manipal Academy of Higher Education, Manipal - 576104, Karnataka, India
3. Department of Ageing Research, Manipal School of Life Sciences, Manipal Academy of Higher Education, Manipal – 576104, Karnataka, India
4. Manipal School of Information Sciences, Manipal Academy of Higher Education, Manipal - 576104, Karnataka, India
5. Department of Pathology, Kasturba Medical College, Manipal Academy of Higher Education, Mangalore - 575001, Karnataka, India
6. Department of Surgery, Kasturba Medical College, Kasturba Medical College, Manipal Academy of Higher Education, Mangalore - 575001, Karnataka, India

*Corresponding author e-mail – [kkmahato@gmail.com](mailto:kkmahato@gmail.com).

Orchid ID: 0000-0001-9873-3445

**Table of Contents**

1. Histology:…………………………………………………………………………………………..….3

2. Photoacoustic spectral data analysis:……………….……………………………………3

2.1 Machine learning:……………………………………...………………………………………….5

3. Liquid chromatography-mass spectrometry (LCMS):……………………………..6

3.1 Metabolomics:……………………………………………………………………..………………..6

4. Histological image processing:………………………………………………….…………11

References:………………………………………………………………………….……………………16

1. **Histology:**

The formalin-fixed tumor tissues were processed as mentioned in materials and methods, and the outcomes have been interpreted in the result section of the main manuscript. As mentioned in the main manuscript, the tumor volume kinetics validated the tumor xenograft in progressive conditions. In contrast, the histological study revealed the structural alterations in the tumor samples belonging to different time points (day 0^th^, 5^th^, 10^th^, 15^th^ and 20^th^ post-MCF7 cell inoculation) under the study. The day 5^th^ H&E staining of tumor sections demonstrated the initial phase of tumor establishment *in vivo*, showing an outer rim of viable tumor cells surrounding the necrotic region in the center with karyopyknosis karyorrhexis. There was minimal vasculature proliferation, indicating initial slow neovascularization. On day 10^th^, viable pleomorphic tumor cells in sheets with slow neovascularization and reduced necrosis was observed. A well-established tumor with well-preserved morphology showing a characteristic of a well-nourished tumor was observed on day 15^th^. Further, due to extreme cellular proliferation and tumor growth, the tumor core becomes hypoxic ^42^. Hence, on day 20^th^, a well-developed vasculature and a necrotic tumor core due to hypoxia were observed. This evidence supports the tumor progression in athymic nude mice of groups from day 5^th^ to day 20^th^.

1. **Photoacoustic spectral data analysis:**

In the present study, the mother wavelet db6 was used as a predictive model because it demonstrated better results than others. The energy distribution of the wavelet coefficients for the level 2 decomposition for the photoacoustic signals of day 0^th^, 5^th^, 10^th^, 15^th^, and 20^th^ groups under study found to be approximately 96%, 96%, 97%, 97%, and 97 %, respectively for approximate coefficient 'AA2' as shown in **Figure S1**. As depicted in Figure S1 and Figure S2, the optimal signal strength was observed in decomposition level 2 under frequency level (2, 0) that is AA2 and hence was considered ideal decomposition level. Since the frequency of interest has maximum energy falling under AA2, it was selected for further analysis.


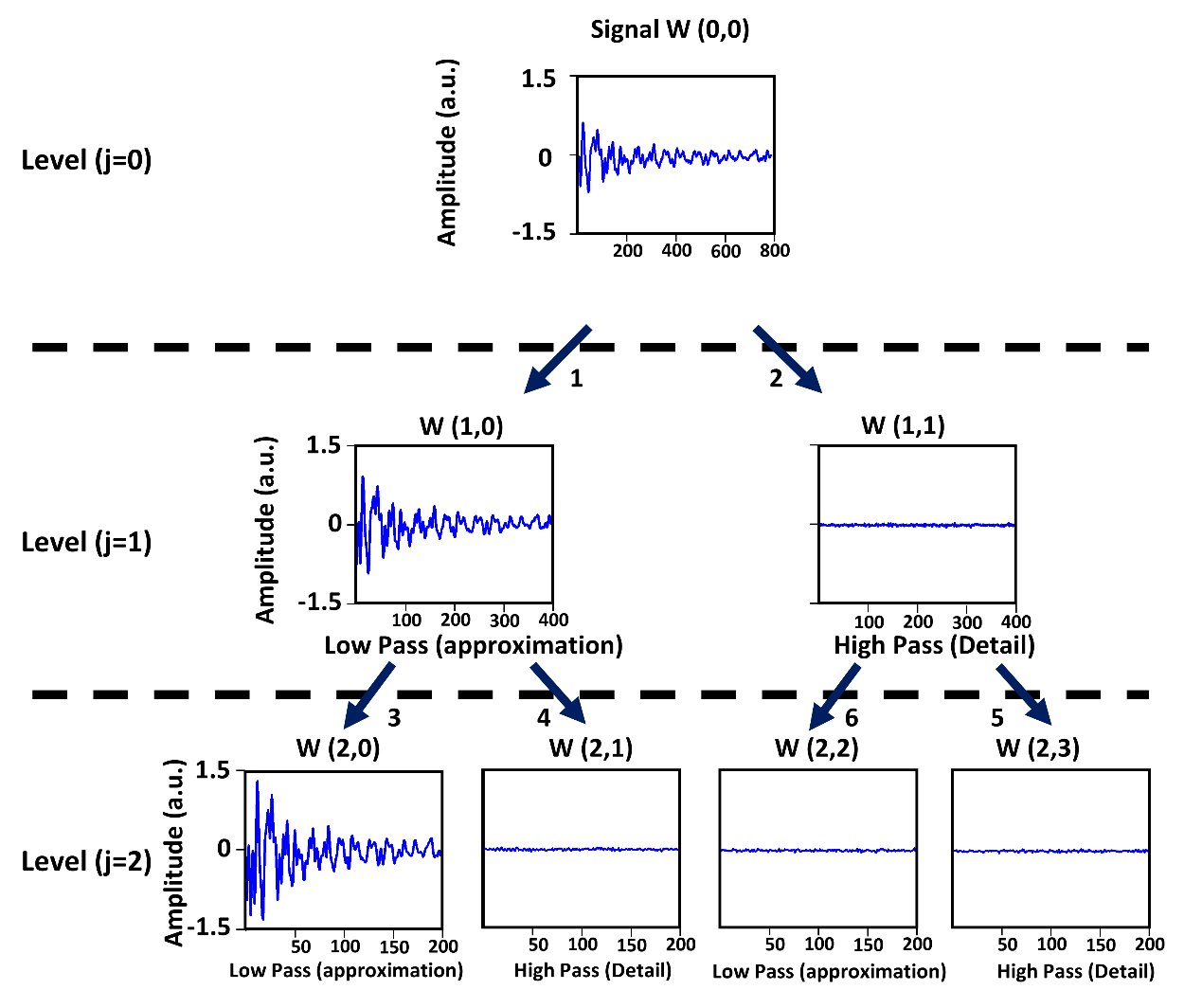


**Figure S1**. The decomposition tree for level 2. The X-axis represents the sample points, and Y-axis represents the signal amplitude

**Figure S2.** Energy Distribution in the coefficients of Level 2 decomposition for different days (0^th^, 5^th^, 10^th^, 15^th,^ and 20^th^) spectra.

*2.1 Machine learning*

The trained SVM learning models were tested for classification of the data, and the obtained performance metrics depicted as a confusion matrix, are shown in Figure S3. The sensitivity, specificity, and accuracy of the classifications by the models were also calculated, as listed in **Table 1** of the main document. The sensitivity values for classification using SVM-RBF, SVM-polynomial and SVM-linear for the 5^th^, 10^th^, 15^th^ and 20^th^ day groups are found to be 98%, 98%, 90.5%, 92.5% ; 100%, 99.5%, 100%, 98% and 98%, 64.5%, 73.5%, 83.5% respectively. Similarly, the specificity and accuracy values for the analysis using SVM-RBF, SVM-Polynomial, and SVM-Linear are 97%, 100%, 92%, and 95.2%, 99.5%, 80.3%, respectively. (Figure S3).


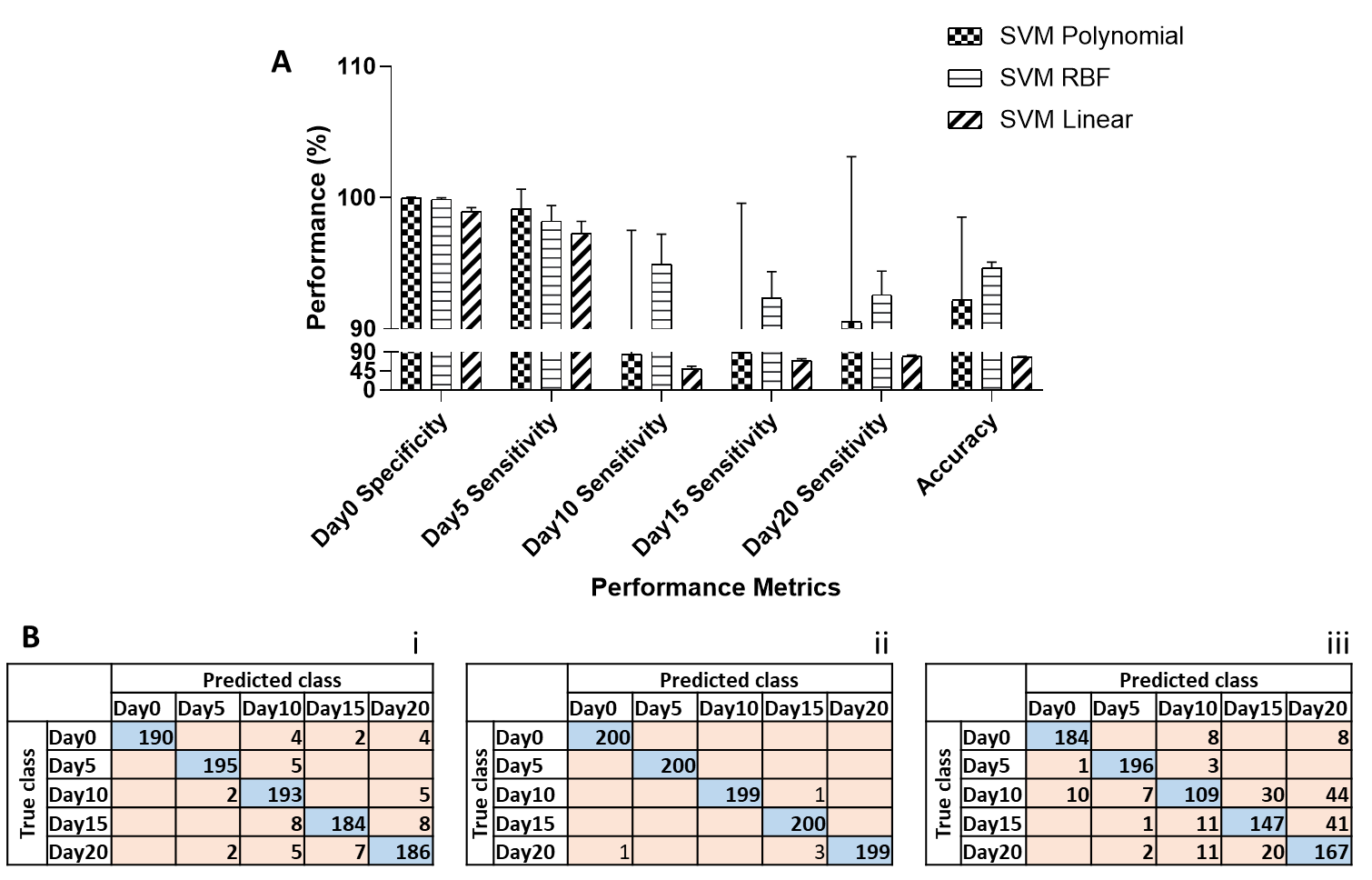


**Figure S3. (A)** Performance measures of SVM analysis for the classification of breast tumor progression. (B) Confusion matrix of the classification for best machine learning models (i) SVM RBF, (ii) SVM Polynomial, and (iii) SVM Linear.

**3. Liquid chromatography-mass spectrometry (LCMS):**

**3.1. Metabolomics**

LC-MS data analysis of the serum samples obtained from the control and test groups under study revealed the presence of a total of 3997 spectral features/metabolites. The data was filtered to their intensity, present in a minimum of 3 out of 5 animals (60%) per group. These metabolites were annotated using the m/z ratio in METLIN and HMDB databases within 15 ppm tolerance. There were 114 compounds annotated, excluding xenobiotics and drugs. Further were used the intensity of the identified compounds to visualize the global metabolome of control and test groups using Circos illustration. The Circos representation showed differential distribution patterns across the control and test groups. The intensity and presence of metabolites were considered in each sample of all the groups. Upon performing 'Student's t-test' and 'One-way ANOVA' on the annotated compounds, 19 metabolites were present in all the experimental groups were altered as shown in **Figure S4**. The significantly altered compounds were subjected to fold change analysis. The fold change was calculated for days 5^th^, 10^th^, 15^th^ & 20^th^ groups compared to the control group, as shown in **Figure S5**.


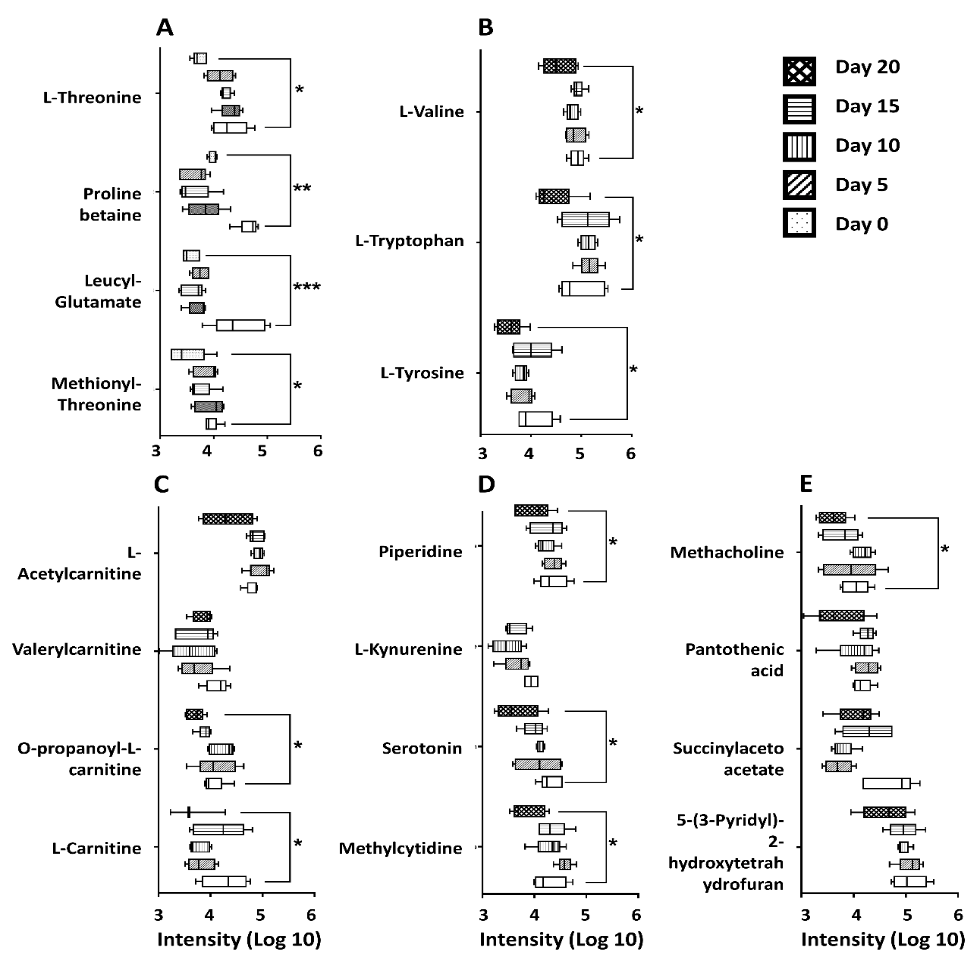


**Figure S5.** Differentially altered serum metabolites in control and test groups. Metabolite intensities of most abundant and significantly altered amino acids (A) and (B), fatty acids (C), and other compounds (D) & (E) detected in serum were log10 transformed and represented as Box-Whiskers plots. Statistically significant changes in metabolite intensity between the control and day 20 group are represented by asterisk (***p < 0.001, **p < 0.01, p < 0.05).


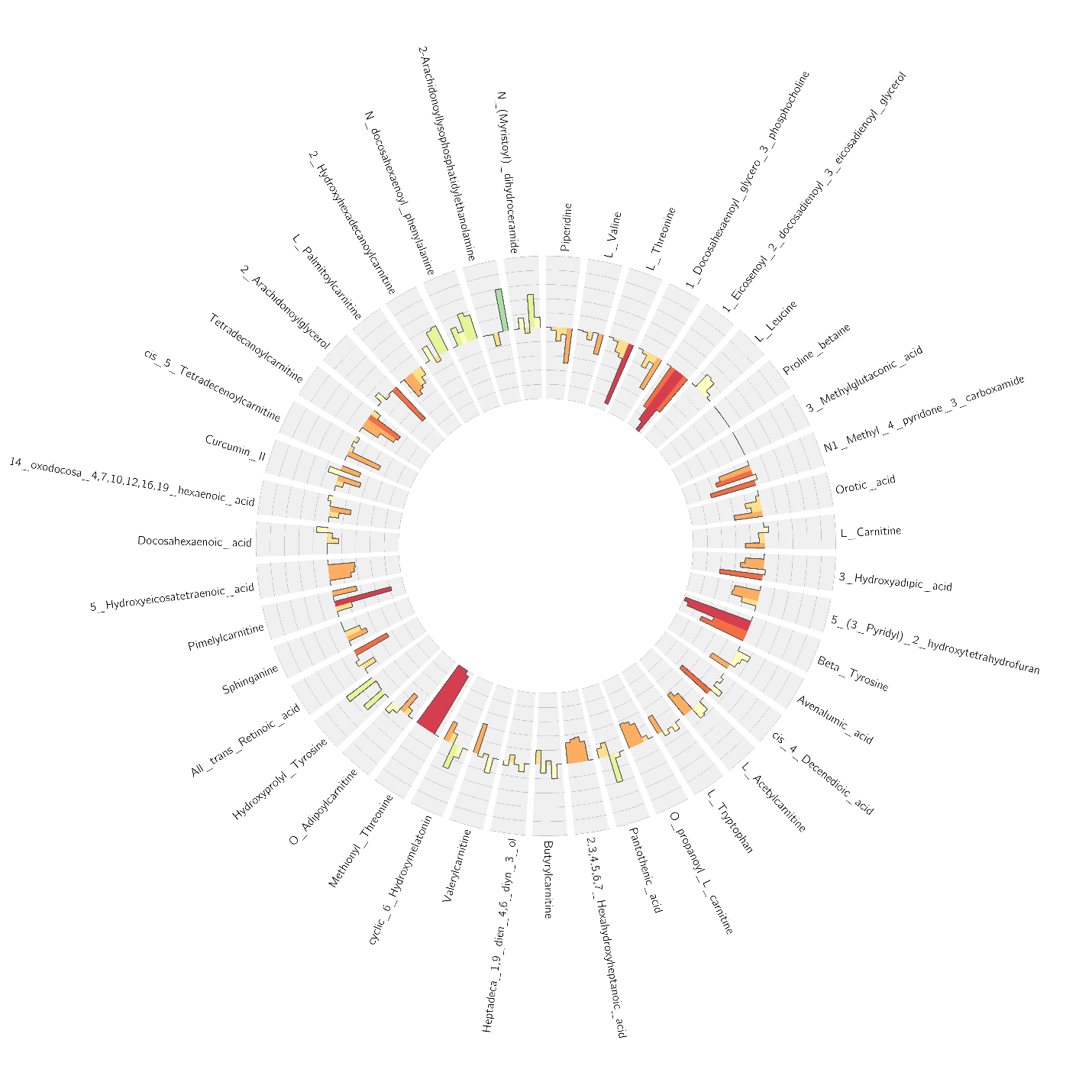


**Figure S5.** Circular bar graph of metabolites using Circos for assessing fold change intensity in each group with respect to control group. The metabolites present in at least 3 animals of each group (day 0^th^, 5^th^, 10^th^, 15^th^ and 20^th^) were considered, and the log10 transformed mean for each sample was used.

The fold change analysis showed that most of the metabolites' serum levels were downregulated as the tumor progressed with respect to the control group. Further, the metabolites from all the time-points were subjected to 'Pearson's correlation analysis,' as shown in the supplementary **Figure S6**.


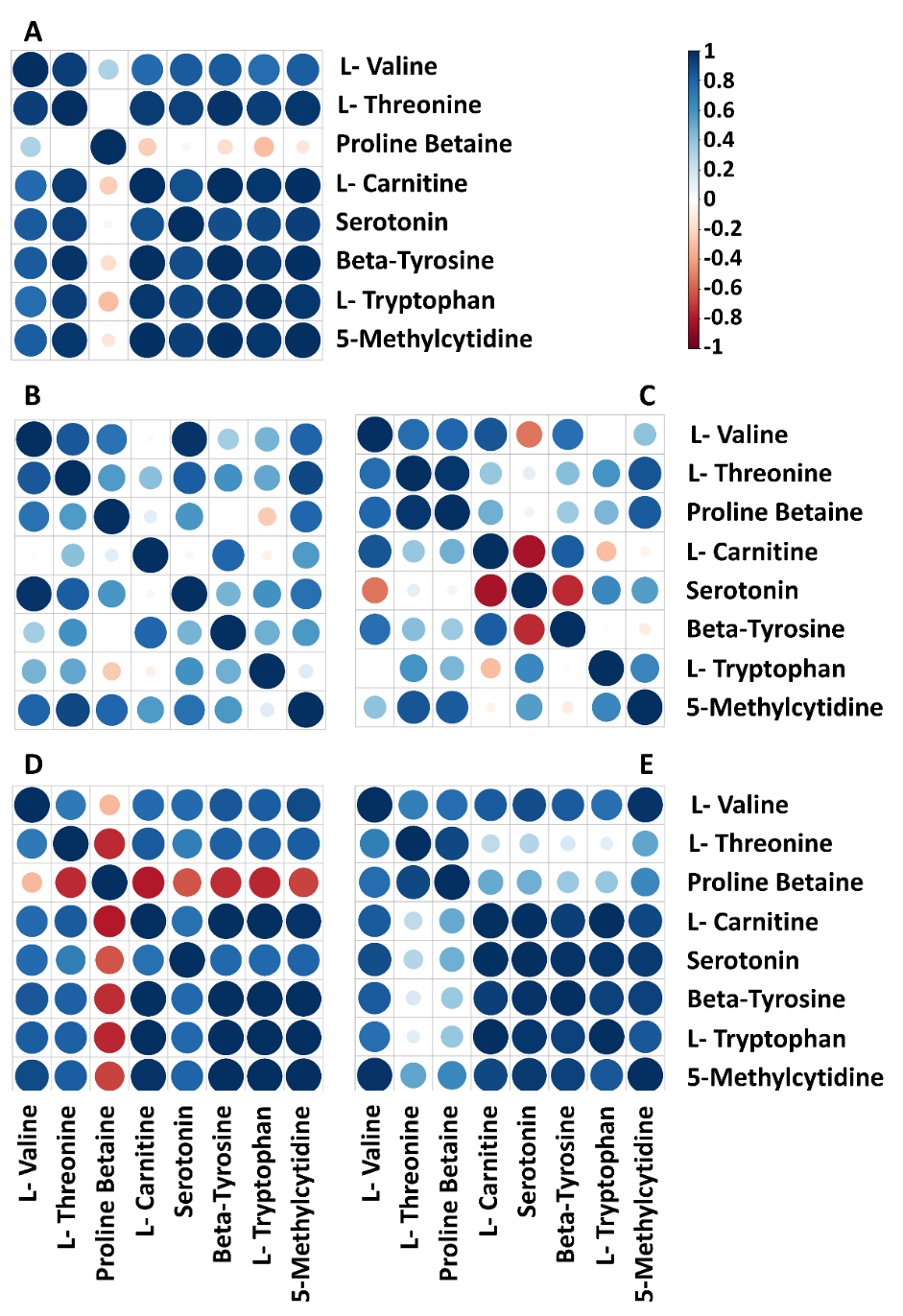


**Figure S6.** Pearson's correlation analysis showing an altered correlation between metabolites present in all samples from day 0^th^ (A), day 5^th^ (B), day 10^th^ (C), day 15^th^ (D) & day 20^th^ (E).

The differentially altered serum metabolites in control and test groups were detected in serum samples based on the most abundant and significantly altered intensities and were transformed to log10 and represented as Box-Whiskers plots. Statistically significant changes in metabolite intensity between the control and day 20 group are represented by asterisk (***p < 0.001, **p < 0.01, p* < 0.05). However, some metabolites showed no statistical significance compared to control.

Among 114 identified compounds, the amino acid levels, lipid levels, and other compounds showed significant variations in the current study. The student's t-test was performed for day 0 versus day 20^th^ group, the amino acids, threonine, proline-betaine, L-valine, tryptophan, tyrosine, and other metabolites such as 5-methylcytidine, serotonin, piperidine and propionyl choline were found to be significantly decreased in progressive tumor condition compared to control. Further, carnitine and o-propanoyl-L-carnitine also showed a similar intensity trend upon tumor progression till day 20^th^. However, metabolites such as acylcarnitine, valeryl carnitine, pantothenic acid, succinyl acetoacetate, and 5-(3-Pyridyl)-2-hydroxytetrahydrofuran had altered intensities upon tumor progression, nevertheless, did not show significant down-regulation.

It is a well-understood fact that the cancer cells undergo extreme metabolic reprogramming, mostly involved in bioenergetic and biosynthetic processes. Mounting evidence has shown rewiring of metabolism in tumor tissues of the breast and its reflection systemically. Metabolomic signatures of cancers can be detected in plasma and identified by metabolomic analysis. Besides metabolites being potential biomarkers of the diseases, they are known to regulate signaling networks or effectors of the disease process itself. Knowledge of specific metabolic pathways will enable the design of therapeutic strategies ^1^. The differentially expressed metabolome was visualized in the present study using CIRCOS illustration, as shown in **figure 7A**. As the tumor progressed, a significant alteration of metabolites was observed using a student t-test.

The metabolomic study revealed a significant decrease in serum tryptophan levels of the day 20^th^ group compared to the control. The altered tryptophan metabolism and the activity of indoleamine 2,3-dioxygenase (IDO) are associated with the pathophysiology of human diseases, including tumor progression ^2,3^. High uptake of tryptophan in the tumor tissues may result in the lesser availability of tryptophan in serum. Tumors exhibit increased uptake of nutrients to meet the requirements of speedily proliferating tumor cells^4,5^. The comparative tryptophan levels in MCF-7 cells are more prominent than non-aggressive tumor cells and normal cells^6^. The pathway analysis in this study using MetaboAnalyst depicted that the tryptophan pathway was among the majorly impacted ones (**Figure 7B)**. The imbalances in the levels of tryptophan and its metabolites have shown an association with a wide variety of human pathologies, including depression, schizophrenia, autoimmunity, neurodegeneration, and cancer ^4^. Further, the optical properties of tryptophan can be used as a fingerprint for monitoring various diseases including breast cancer^7^. These studies served as a valuable evidence to support our machine learning based photoacoustic study by targeting tryptophan of tumor tissues to obtain differential photoacoustic signals from them of different time point groups.

1. **Histological image processing**

Image processing on the 125 H&E stained histological photomicrographs at 400x, 25 belonging to each group (Day 0^th^, 5^th^, 10^th^, 15^th^ & 20^th^) understudy was accomplished using TissueQuant software. The H&E stained slides were evaluated by an expert histopathologist to select the region of interest and capture the image at 400x magnification containing features including viable tumor cells, necrosis, growth of blood capillaries, and RBCs entrapped.

The histological features like necrosis, viable tumor cells, growth of blood capillaries, RBCs entrapped, etc., were quantified in terms of pixel color scores of the H&E-stained histological images at 400x magnification by TissueQuant software. The TissueQuant software is a Java-based software publicly available color quantification tool (website: <https://manipal.edu/sois/research/TQ_Tool.html>), used to quantify the histological features of the progressing tumor xenograft in athymic nude mice. Initially, under a pathologist's supervision, the color ranges in H&E stained histological images were detected and quantified using the software based on tumor tissue staining properties. The scores obtained through the software quantifies the biological features based on the color intensities present in the image under study ^8^. To conduct this analysis using TissueQuant software, H&E stained histological images of different groups understudy in 400x magnification were chosen. This was followed by selecting the region of interest (ROI) in the image. Upon clicking on the ROI, the software calculates hue, saturation, and intensity values of the selected color in the image by considering the corresponding centers of Gaussian weighting functions. Each pixel's scores in the 0 - 255 range were then plotted to display the color of interest as a grayscale image (**Figure S7A**). Along with the grayscale images, the software will also display the mean color score and the number of pixels representing the image's area with the selected color shades. The score and the area were multiplied to obtain the intensity of the chosen color shade, which is then plotted using GraphPad Prism 8.0 (**Figure S7B**).

The intensity scores of 3 features selected for the quantification based on the color range in the ROI of the image are necrosis, blood, and cells and plotted using GraphPad prism 8.0, as shown in figure 4B. In control, the normal intensity for cells and blood vessels was noticed, and a negligible intensity for necrosis was observed. In the experimental data, it was observed that the intensity of cells has gradually increased from day 5^th^ to day 10^th,^ and after that started decreasing as other features increase. In the case of blood capillaries, it kept rising from day 5^th^ till day 20^th^. However, the necrosis was observed more on day 5^th^ than any other group, and as the time point progressed, the necrosis was observed less and again persisted on day 20^th^.


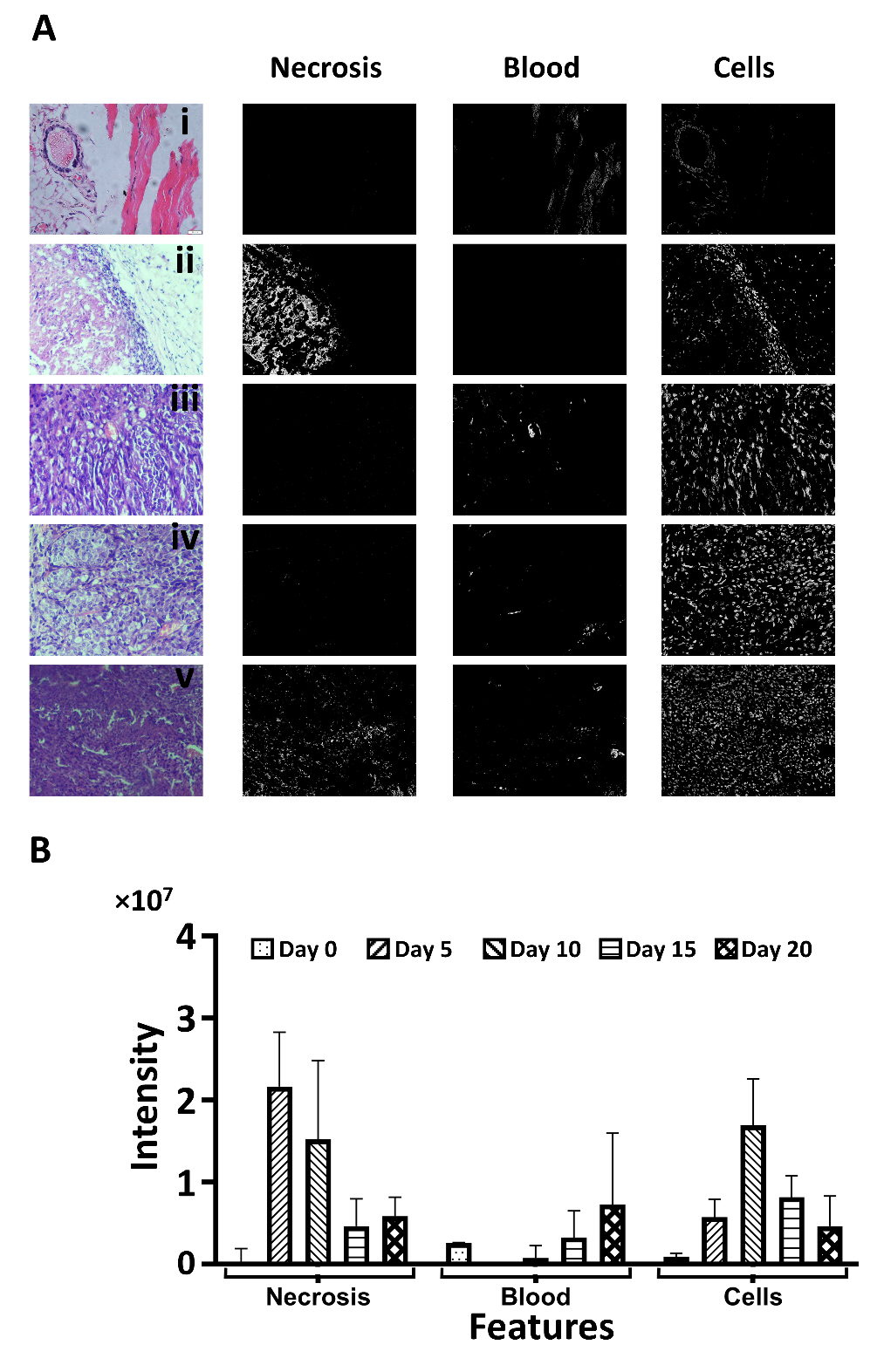


**Figure S7.** (A) Typical processed images of H&E stained photomicrographs in 400x magnification of different days (0^th^, 5^th^, 10^th^, 15^th,^ and 20^th^) of tumor progression in the left panel (i-v) and the features like Necrosis, Blood capillaries, and Cells extracted from them using the TissueQuant software. (B) Quantitative analysis of Necrosis, Blood, and cells in H&E stained photomicrographs at 400x magnification of different days (0^th^, 5^th^, 10^th^, 15^th,^ and 20^th^) of tumor progression by TissueQuant software.

Further, histological image processing has become a powerful scientific tool in measuring the optical parameters and color characteristics in the image. The technique objectively assesses image data and is convenient for analyzing large image datasets with reduced human errors compared to subjective evaluations. Immunohistological sections of breast cancer and normal counter tissues have been classified accurately through image processing^9^. Lehr HA et al. have shown the application of a simple, inexpensive Adobe Photoshop based algorithm in routine diagnostic pathology for quantifying Ki-67-expression in breast cancer using custom-made plug-ins applied on Photoshop-based image analysis^10^. In the present histological image processing, the pathological feature, blood capillaries were identified by the TissueQuant software based on the reference color range of RBCs entrapped in the capillaries, as the time points progressed. The feature scores were increasing as the time point increased from day 10^th^ till day 20^th^. In the control group, the blood was found entrapped in the normal breast vessels, and no blood was seen in day 5^th^ images. This finding of blood capillaries by the TissueQuant software matched well with the histological evidence of tumor progression (**Figure S7B**). Further, the cells were identified by the software in the slides with the hematoxylin stains. In principle, a dye with basic nature shows an affinity towards acidic components, whereas that with an acidic environment binds to basic components. In hematoxylin and eosin staining, hematoxylin (blue) stains to the cells' nucleus and the eosin (Pink), the cytoplasm of the cells. For other cellular structures, the H&E in combination taking on different shades, hues, and combinations of these colors. The stain showed the general layout and distribution of cells and provided a standard overview of the tissue sample structure under study. Hence, a pathologist can easily differentiate between the nuclear and cytoplasmic regions of a cell. This primary information was incorporated in the TissueQuant software to select features from the histological slides under study. As per histological data, there was increased angiogenesis in the slides belong to day 15^th^, evident from the increased number of blood capillaries and extensive necrosis in some regions of the images and still greater angiogenesis in day 20^th^ images were observed. The score value for cells of day 0^th^ was found to be contributed by the epithelial cells present in the formation of the duct of the normal breast in nude mice. Usually, the cells are intact when they are healthy and proliferating, but it would rupture the nuclear content and spill out the DNA as they undergo stress. This makes the H&E stained histological sections appear pink without blue or irregular traces of blue. This is the necrosis and mentioned in the histological analysis as well and image processing by TissueQuant software. The image processing reveals that the necrosis was more on day 5^th^ and was increased till day 15^th,^ and thereafter, an increase in the score values for the day 20^th^ group was observed. This clearly suggests that the necrosis has increased on day 20^th^ and hence its score values. The score value was found almost nil for the day, 0^th^ group. The shades of eosin in some regions of day 0^th^ slides were noticed because of the H&E stained color range of muscles is close to that of necrosis, as reflected in the graph (**Figure S7B)**. This data also correlates histological findings as the extensive necrosis was found on day 5^th^ on establishing tumor cells *in vivo*. On day 15^th^, a well-established tumor microenvironment with minimum necrosis and necrosis was observed to be increased due to the aggressive nature of the tumors that outgrown the blood capillaries during aggressive proliferation leading to tumor hypoxia increasing necrosis^11^.

**References:**

1. Hart, C. D., Tenori, L., Luchinat, C. & Leo, A. Di. *Novel Biomarkers in the Continuum of Breast Cancer*. *Springer, Cham* **882**, (Springer International Publishing, 2016).

2. Gasparri, A. M. *et al.* Critical role of indoleamine 2,3-dioxygenase in tumor resistance to repeated treatments with targeted IFN. *Mol. Cancer Ther.* **7**, 3859–3866 (2008).

3. Uyttenhove, C. *et al.* Evidence for a tumoral immune resistance mechanism based on tryptophan degradation by indoleamine 2,3-dioxygenase. *Nat. Med.* **9**, 1269–1274 (2003).

4. Sordillo, L. A., Pu, Y., Ph, D. & Budansky, Y. Optical Spectral Fingerprints of Tissues from Patients with Different Breast Cancer Histologies Using a Novel Fluorescence Spectroscopic Device. **12**, 455–461 (2013).

5. Venkateswaran, N. *et al.* MYC promotes tryptophan uptake and metabolism by the kynurenine pathway in colon cancer. *Genes Dev.* **33**, 1236–1251 (2019).

6. Zhang, L. *et al.* Tryptophan as the fingerprint for distinguishing aggressiveness among breast cancer cell lines using native fluorescence spectroscopy. *J. Biomed. Opt.* **19**, 37005 (2014).

7. Sordillo, L. A., Pu, Y., Sordillo, P. P., Budansky, Y. & Alfano, R. R. Optical Spectral Fingerprints of Tissues from Patients with Different Breast Cancer Histologies Using a Novel Fluorescence Spectroscopic Device. *Technol. Cancer Res. Treat.* **12**, 455–461 (2013).

8. Prasad, K., Kumar P., B., Chakravarthy, M. & Prabhu, G. Applications of ‘TissueQuant’– A color intensity quantification tool for medical research. *Comput. Methods Programs Biomed.* **106**, 27–36 (2012).

9. Kumar, A., Rao, A., Bhavani, S., Newberg, J. Y. & Murphy, R. F. Automated analysis of immunohistochemistry images identifies candidate location biomarkers for cancers. *Proc. Natl. Acad. Sci.* **111**, 18249–18254 (2014).

10. Lehr, H.-A., Mankoff, D. A., Corwin, D., Santeusanio, G. & Gown, A. M. Application of Photoshop-based Image Analysis to Quantification of Hormone Receptor Expression in Breast Cancer. *J. Histochem. Cytochem.* **45**, 1559–1565 (1997).

11. Bredholt, G. *et al.* Tumor necrosis is an important hallmark of aggressive endometrial cancer and associates with hypoxia, angiogenesis and inflammation responses. *Oncotarget* **6**, 39676–39691 (2015).
